# Medical Items and News of Interest to the Physician

**Published:** 1879-07

**Authors:** 


### For the Use of Physicians.

CULLED FROM THE STANDARD MEDICAL JOURNALS OF THE WORLD.

NOTE.—These formulæ are intended only for the regular practitioner of medicine, and should be employed only by him, or under his immediate supervision.

**To Arrest Vomiting During Pregnancy.**

℞ Cerii oxalat.,
Ipecacuanhæ, āā gr. i.
Creasoti, gtt. ij. M.
Sig. To be taken every hour.

**Chloral Liniment** (*Catillon.*)—

Hydrate of chloral, 6 parts.
Oil of sweet almonds, 30 parts.

Dissolve by rubbing in a mortar, or by mixing them in a flask and immersing the latter into hot water.

**Chloral Ointment** (*Catillon*).

Hydrate of Chloral, 6 parts.
Lard,.............27 "
White wax........ 3 "

Melt the lard and wax in a beaker placed into hot water, add the chloral in powder, and dissolve. Then let cool.

**Ointment for Piles.**

℞

Pulv. opii, ʒ ss.

Acid tannici, ʒ i.

Acid carbolici, gr. xv.

Ol. tabaci. ♏ x.

Liq. plumb. subacet. ♏ xx.

Unguenti simpl. ℥ i.

Mix intimately. To be used night and morning.

**Fothergill's Cough Mixture.**

Numerous trials have been made in the wards of the Episcopal Hospital, in Philadelphia, by Dr. Bennet, of Dr. J. Milner Fothergill's boasted cough mixture, from the use of which he claims such excellent results, viz:

℞
Acid. hydrobrom, gtt. xx.
Ext. scillæ, gr. ss.
Spts. chloroformi, f. ℥ ss. M.
Sig. Every three hours.

The experience of the attending physician has been that it does not at all take the place of morphia, as claimed by its originator.—*Med. Rec.*

**Lotion for Sore Nipples.**

℞

Sodii boratis, ʒ ij.

Cretæ præcip, ʒ i.

Alcoholis, fl. ℥ ij.

Aquæ, fl. ʒ ij.

Mix; shake well before applying.—*The Practitioner.*

**Salicylic Acid against Tænia.**

After trying almost all other remedies in vain, Marynowski administered to a lady who had suffered with tænia solium for nine years, 0.5 salicylic acid four times, at intervals of one hour, and then gave a tablespoonful of castor oil. This treatment proved painless and perfectfully successful.—*Apoth. Ztg.*, *Jour Ph.*

**Chloroform Narcosis.**

Wachsmuth, of Berlin, asserts that much of the danger from the administration of chloroform may be averted by adding to it 20 per cent. of oil of turpentine, which, he says, stimulates the lungs and thus protects them against the great enemy of chloroform narcosis—pulmonary paralysis.—*Louisville Medical News.*

**Eucalyptus for a Cold in the Head.**

Prof. Strambio, in a note in the *Gaz. Med. Ital. Lombard*, says that, notwithstanding the failure of all the remedies hitherto recommended for the immediate cure of a cold, he wishes to communicate to the profession the great success he has found attending a new one in his own person, and to ask them to test its efficacy. He found prolonged mastication and swallowing of a dried leaf or two of the *Eucalyptus globulus* almost immediately liberated him from all the effects of a severe cold.

**Sedative Action of Quinia on the Neck of the Bladder.**

Dr. Simmons, of the Ken Hospital, Yokohama, Japan, observes that the relief obtained in some painful affections of the bladder by injecting quinia into the viscus is generally known; but lately he discovered accidentally that large doses of from ten to fifteen grains acted as beneficially when taken by the mouth in a case of painful and persistent tenesmus of the organ. In two other severe cases it has been tried with the same benefit. This sedative action, he suggests, may explain the action of quinia in preventing urethral fever when administed prior to operations on the part.

**Hypodermic Injection of Salicylic Acid in Erysipelas.**

Prof. Ferdinand Peterson, of Kiel, states that he has thrice arrested the progress of erysipelas by injecting a concentrated solution of salicylic acid under the healthy skin surrounding the diseased part. Several such injections were made simultaneously in each of the cases. He does not, however, positively assert that the good effect was simply owing to the operation.

---

**Phosphorus in Sciatica.**

Dr. Volquardsen, in a Pesth medical journal quoted by the London *Medical Record*, reports a case of sciatica, which lasted for two years and defied all treatment. He then arrived at the idea of trying the internal use of phosphorous, which he prescribed in doses of fifteen milligrams (about one-fourth of a grain) three times a day. Three days sufficed to obtain a marked improvement, and three weeks brought a complete cure.

---

**Chloroform.**

Death from chloroform need never occur, according to the doctrine of Syme, Lister and Hughes, if this simple rule be observed: "Never mind the pulse, never mind the heart, leave the pupil to itself. Keep your eye on the breathing; and if it becomes embarrassed to a grave extent, take an artery-forceps and pull the tongue well out." Syme never lost a case from chloroform, although he gave it five thousand times; this simple rule enabled him (so he thought) to make this excellent record. —*Lancet.*

---

**Effect of Rhubarb in Preventing Nausea from Morphia.**

Dr. R. H. Tatum, of Dayton, Va., believes that when a few grains of *powdered* rhubarb are given with morphia it will not only prevent nausea through the tonic effect of its action on the gastric mucous membrane, but will also improve the appetite, even when given nightly for a long period of time. On the omission of the rhubarb he has observed that the unpleasant effects of the morphia become manifest.—*Virginia Med. Monthly.*

---

**New Mode of Using Ether as an Anæsthetic.**

Dr. Packard (*Phil. Med. Times*, December 21st, 1878,) recommends for operations not lasting over forty seconds, the following procedure: Let the patient lie upon the sofa, and take a sponge wet with ether in his own hand, directing him to hold the other arm up in the air. After breathing the ether for a few moments, the arm will drop and you will have from thirty to forty seconds of unconsciousness in which to operate. The sponge is removed and the patient is ready to go about his business. This procedure gives rise to no headache, nausea, or other unpleasant symptoms, and is particularly useful is children. The chief source of disappointment is in not recognizing the right moment; for if this be allowed to pass, unconsciousness will not again occur until full etherization. When the arm wavers, be ready, and as soon as it drops, perform the operation, and there will be no pain felt.

---

**Nitrate of Pilocarpine as a Remedy in Pleuro-pneumonia.**

In a case of pneumonia with pleurisy in a man of 55, Dr. T. H. Newland, of St. Louis, gave $\frac{1}{8}$ gr. of nitrate of pilocarpine twice daily for three successive days, giving it hypodermically.

Its sialagogue effect came on in about 25 minutes, and diaphoresis about 10 minutes later, the patient being each time greatly relieved as regards pain, sleep, ability to lie down, and ease of respiration. Poultices to the chest were also employed.—*St. Louis Med. and Surg. Jour.*

---

**Zinc Oxide in Diarrhœa.**

Dr. Jacquier has tested the good effects of the employment of oxide of zinc in diarrhœa. He administers every six hours a powder consisting of

Oxide of zinc, 14 gr.
Sodium bicarbonate, 2 gr.

In some cases the malady had existed from one to many months, and other methods of treatment had not produced any improvement. In all cases which were treated in this manner by a colleague of his, there was only one in which three doses of the remedy were required.—*Brit. Med. Journ.*

---

**Cactus and Heart's Ease.**

Cactus and heart's-ease act on the heart, and are useful in heart disease. Lungwort acts on the lungs, and is useful in lung disease. Tincture of arnica, in very small doses, will stop bleeding at the lungs. Prussiate of potash will cicatrize the lungs, if ulcerated, and relieve the cough. Balsam of tolu is healing to the lungs, and when combined with tincture of myrrh, syrup of senega and squills, will cure almost any cough that is curable.—*Toledo Medical Journal.*

---

**Aromatic Liquid Pepsin** (*Biroth.*)—

Saccharated pepsin, 16.5 parts or grams.
Hydrochloric acid, 3.7 parts or grams.
Glycerin, 177.1 parts or grams.
Orange flower water, 236.0 parts or grams.
Bitter almond water, 2.0 parts or grams.

Dissolve the pepsin in the aromatic waters, to which the acid has been added; then add the glycerin. Dose: ½—1 teaspoonful for children; a tablespoonful for adults.—*L'Orosi.*

---

**Chloral Suppositories** (*Catillon.*)

1. Containing 1 gram (15½ gr.) each:
   Hydrate of chloral, 1 gram.
   White wax.........1 "
   Cacao butter.......3 grams.
2. Containing 2 grams (31 gr.) each:
   Hydrate of chloral, 2 grams.
   White wax........1.5 "
   Cacao butter......2.5 "

Melt the wax and cacao butter in a beaker or test tube, add the chloral in powder. When dissolved pour into moulds.—*Rep. de Pharm.*

---

**For Chronic Bronchitis.**

The administration of remedies by inhalation commends itself to the judgment as being a ready way of getting directly at the seat of the disease. One writer, of Boston, declares in a medical journal that he had found a solution of carbolic acid (at 2½ percent., raised afterward to 5 per cent.) the only efficacious treatment he had ever used. It soon cured him permanently of a winter cough that had distressed him for many years. A late number of the Philadelphia *Medical Times*, contains a communication from Dr. Robert Fletcher relating some of his experiences with atomized chloral. A lady of 50 had a severe racking chronic cough, which had troubled her for several successive winters. All other remedies having failed, some being in the atomized form, a solution was prepared of ten grains of chloral to the ounce of water, which was inhaled in a steam atomizer night and morning. Improvement soon occurred, and after two weeks' use of the remedy the cough had entirely disappeared, and had not returned since. In a second case there was a similar result.

**Compound Bismuth Powder.**

In answer to a query relating to the above, a correspondent from Portland, Me., suggests that the preparation is in all probability an article kept in all the drug stores there, and prescribed under that name by most physicians. It is made as follows:

| | | |
|---|---|---|
| Subnitrate of bismuth, | 1 | ounce. |
| Bicarbonate of soda, | 1 | " |
| White sugar, powdered, | 1 | " |
| Jamacia ginger, " | 1 | " |
| Gum arabic, " | 1 | " |

Mix together.

—*Druggist's Circular.*

---

**Bromide of Ethyl in Neuralgia and Sleeplessness.**

Winckel and Fiedler (*Allg. Med. Cent. Ztg.*) say that this preparation, the properties of which are similar to those of chloride of ethyl, was first used in France in cases of carcinoma with neuralgia and sleeplessness. Dr. Winckel observed good results from its use in neuralgia, of myoma and beginning epithelioma. The hypnotic effect was unmistakable. The dose of bromide of ethyl is 10 to 15 to 20 drops in water before going to bed. It is easily taken. Dr. Feidler adds that he has used this substance in eight cases of marked insomnia, having given it in the doses just mentioned. Two of the patients who had long suffered from sleeplessness slept well afterwards. The number is, of course, too small, excepting as it seems to invite further trial of the remedy.—*Phil. Med. Times.*

---

**Hemp Smoking in Tetanus.**

Mr. Khastagir has successfully treated five cases of traumatic tetanus under the effect of ganja (hemp) smoking. From the insufficiency of the number of doses ordered, since the effect wears off in the interval; or from the drug being used as an auxiliary to some more potent remedy, in which case its effect is unnoticed, it is administered by causing an attendant to keep a smoking-pipe ready near the patient, charged with about 15 grains of the dried leaves, either alone or mixed with twice the amount of dried tobacco leaves. On every reappearance of a clonic spasm, the patient is made to smoke the pipe till the leaves are burnt to ashes, on which the muscles of the body instantly relax, the patient shuts his eyes and apparently falls asleep. The attendant again charges the pipe with the dried ganja leaves, and watches the patient for the advent of the next spasm to again make him smoke. In this way the drug has been administered day and night uninterruptedly, during which the irritation of the nervous system slowly, but steadily, yielded to the effect of the drug. The longest time which the hemp takes to cure the disease has—according to the author's observations—been six weeks, the shortest seven days. The only auxiliary medicine that need be used in the course of the treatment, is an occasional dose of purgative mixture by the mouth, or enema by the rectum to relieve constipation. In one case a dose of hydrated chloral was required to be given at night to produce sleep. Milk and soup are the only nutriments which the patient can or should take, until he is able to masticate or swallow solid food, without giving occasion to fresh attacks of clonic spasms.—*Indian Medical Gazette.*

---

**Benzoate of Lithia.**

Dr. T. O. Summers: "In our hands benzoate of lithia has often a most magical effect in diminishing the uric acid deposits and increasing the free hippuric acid of the urine. It will thus be seen that this agent acts in a manner entirely different from the alkalies, in the cure of uric acid deposits. Benzoate of lithia acts upon the urine before it leaves the blood, converting the uric acid, which would otherwise be deposited, into hippuric acid, a harmless agent, which passes off in solution, leaving no ill effects. The alkalies do not act in this way. They dissolve the uric acid directly after it has left the blood. Hence, while they are clearly indicated in those cases in which the acidity of the urine is so great as to produce irritability of the bladder or any part of the genito-urinary apparatus, it is clear that they cannot change the lithic acid diathesis—an end which we may hope to attain according to the rationale of the benzoate of lithia action."—*Nashville Journal of Medicine and Surgery.*

---

**German Treatment of Croup and Diphtheria.**

The following treatment of croup and diphtheria is advocated by Dr. Taube, in the *Deutsche Zeitschrift fur Prakt. Medicin*, September, 1878. An ordinary inhaler is to be taken, full of water, into which is to be put each time it is used fifteen drops of the oil of turpentine. The child should be wrapped in a sheet and placed on its mother's lap, while another person holds the apparatus for eight or ten minutes, about three inches from its mouth. It is well to grease the child's face and to protect the eyes with a cloth. At first the inhalation should be practiced every hour, day and night, the favorable result of which will soon be apparent. With regard to the carbolic acid injection, it should be made into the submucous tissue, half the contents of an ordinary hypodermic syringe, containing a weak solution of the acid (3 to 1,000) being injected two or three times a day into the tonsils. Subject to certain modifications, as the case may require, Taube would recommend the following plan: 1. Hourly inhalations of the oil of turpentine throughout the day and night. 2. Injections of carbolic acid two or three times a day. 3. From one to two teaspoonfuls of Port wine or maderia, to be given every hour; cold compress to the neck; two or three times a day a warm bath with the cold affusion; and small doses of the infusion of digitalis, to which a little benzoic acid is added. The nourishment should consist of eggs and milk only. Constipation is to be met with linseed oil, and for the separation of the false membrane, sulphate of copper, or tracheotomy, with turpentine inhalations through and above the canula.—*Medical and Surgical Reporter.*

---

**Capsicum and Quinine.**

Capsicum combined with quinine will diminish the size of the dose requisite, and the same may be said of ginger and other aromatics. A good dose of capsicum combined with twenty grains of quinine will act as well as thirty grains of quinine without the capsicum. Spices in general stimulate the portal circulation, and promote the flow of bile, and hence their universal use in hot climates. There is a tendency on the part of quinine and capsicum to purge, and sometimes to purge violently. In such cases the purgative action is caused by the increased flow of bile produced by the capsicum. Ginger and quinine when combined do not purge, and it makes a very good combination. If the medicine is administered in form of pills, capsicum may be preferable, because of the less bulk required; but if desirable, the ginger may be given separately, and with the same effect as when combined with the quinine. The proportions should be one grain of capsicum to three grains of quinine; with ginger one grain of each.—*The Medical Brief.*

**Damiana.**

For a long time we found no patient who seemed a proper subject upon which to test it. At last, our friend, Dr. de Cailhol, described to us a case, that seemed a good one to test the alleged virtues of damiana upon. We furnished the doctor with two bottles, which he gave to his patient with directions to take one teaspoonful three times a day. The case was one of complete impotency following an attack of malarial fever. This had resisted phosphorus, taken for some time, also nux vomica and strychnia. The patient completely recovered from his affection before he had finished taking the amount named, and now, two months afterward, is fully restored.

We have another case under observation and hope to report it in a few months. We are satisfied that the drug has a special aphrodisiac effect; how far it may be available for medical purposes is, as yet, undetermined.—*St. Louis Clinical Record.*

---

**Caffeine as a Diuretic and Cardiac Stimulant.**

Some time ago Prof. Gubler stated that caffeine induced abundant and instantaneous diuresis in cases of cardiac dropsy. Dr. L. Shapter has recently reported cases which confirm this observation, and indicate a stimulating action of the drug upon the heart. In these cases the patients were in the latter stages of heart disease, with dropsy, dyspnœa, weak and irregular heart action, and diminished renal excretion. Caffeine, in gr. iij doses from one to three times a day, was given, after digitalis and potash had been tried unsuccessfully. All of the four reported cases showed rapid and marked improvement the urine generally doubling in amout within twenty-four hours.

Caffeine has been shown to be a vascular and cardiac tonic. Whether in these weak and dilated hearts it acts as a direct stimulant or chiefly by increasing diuresis and thus unloading the circulation it is impossible to say, but at any rate, its special value seems to be pretty well shown.—*The Practitioner.*

---

**Eczema of the Head.**

Dr. Bulkley, of this city, presented the following case at his clinic: When you first saw this man it was a case of acute erythematous eczema (*eczema rubrum*), and in some portions the eruption, you remember, was very moist. It was greatley aggravated just behind the ears, and the ears themselves were stiff and in a state of fixation, appearing almost as if they would break off if handled. Now, on the contrary, the moisture has entirely left the eruption, which has dried up to a great extent, and the ears are perfectly suple and movable. He is taking what is known in my clinic as Startin's mixture, of sulphate of magnesia and iron, after the following formula:

℞

Magnes. sulphat, ℥ j.

Ferri sulph., ʒ ss.

Acidi sulph., dil., ʒ ij.

Infus. quassiæ, ℥ viij. M.

Sig.—Take two teaspoonfuls after eating, in water, through a tube. Locally he at first found most relief from oxide of zinc ointment (ʒ j.–℥ j.); he now is rapidly improving under that of tannin (ʒ j.–℥ j.) *Rosevelt Hospital Rep.*

---

**Diphtheria and Chlorine Water.**

Dr. Washburn, of Rensselaer, Indiana, has written a letter to the *Med. and Surg. Reporter* of Philadelphia, strongly recommending chlorine for this affection. He says:

"I have used chlorine water since the summer of 1872, and would not exchange it for any other single remedy that I have ever used or seen used. I cannot claim that it will cure every case, and yet I might, were I to make my diagnosis in accordance with my success, as some do. In that case I would call all fatal cases membranous croup, croup and diphtheria, or malignant tonsillitis.

"I am certain that I do not know any remedy that will cure every case of diphtheria. The following is the formula I use in the preparation of chlorine water:

℞

Chlorate of potassium, gr. xx.
Hydrochloric acid, ♏ xx.
Water, ℥ iv. M.

Prepared as follows: Pulverize the potash and put in a dry vial, then add the acid, corking the vial, to retain the chlorine set free by the reaction. After that has ceased, add an ounce or two of the water, agitate until all the chlorine is dissolved in the water, then add water to make it the desired strength. Dose, teaspoonful every two or three hours, as required.

"It ought to be of a green color, and it will lose that if not kept in a dark colored bottle, wrapped in colored paper, or kept in a dark place. The medicine ought to be freshly prepared every second or third day."

---

**Milk Diet in Inflammation of the Bladder.**

The remarkable success of milk as an exclusive article of diet in this form of malady is noticed in a recent number of the London *Lancet.* Mr. W. Teevan gives an account of a man on whom he had successfully operated for stone, but who some years afterward became a great sufferer from chronic cystitis. He was always in pain, and his urine was very thick. His nights were broken and his strength greatly lessened, and he was not able to work. No treatment gave any relief. Under these circumstances, says Mr. Teevan, I determined to try Dr. Johnson's method of an exclusively milk diet. Having put his alimentary canal in a fit and proper condition for commencing the treatment, the patient began on June 20th to live on milk alone, taking half a pint every two hours, his urine being then a mass of muco-pus, which adhered tenaciously to the *pot de chambre.* He could not pass any urine without the catheter, and was always worried by a dull aching pain above the pubes and in the rectum. The next evening he complained of an acid taste in his mouth, and brought up several pieces of curdled milk.

When there was a tendency of the milk to curdle the patient was given bismuth. He progressed rapidly, and on July 5th was discharged cured of his cystitis, and a month afterward he remained quite well, but had, of course, to use his catheter as previously, on account of an enlarged prostate.

---

**Surgical Treatment of Anasarca.**

Dr. H. A. Wickers writes to the *Lancet:* The treatment of anasarca, whether cardiac or renal in its origin, after ordinary therapeutic measures have failed, is in most instances very unsatisfactory. If the patient be let alone, after a time he gets water-logged and dies; incisions nearly always lead to sloughing; and other plans of treatment, such as drainage, tubes, etc., have proved scarcely less unsuccessful.

The following method has been in use for several months at Charing-cross Hospital, where it has been employed in some forty or fifty cases; it is readily carried out, gives great relief to the patient, and has never been followed by ulceration, sloughing, or inflammation.

The legs having been well oiled, and a mackintosh sheet placed under them, about twenty or thirty punctures are rapidly made in their sides with a stout needle or hare-lip pin; some sponges which have been well wrung out in a saturated, watery solution of salicylic acid, are now placed against the punctures so as to absorb the fluid as it transudes; these sponges as they become filled are squeezed out, and again passed through a solution of salicylic acid before being replaced against the patient's skin. In this manner renewals may be required about every two or three hours; and four or five pints of fluid may be drained away during the first day, the whole process being possibly completed in four or five days, at the end of which time the punctures are usually healed. By the use of salicylic acid decomposition of the dropsical fluid does not occur, the sponges are kept free from fetor, the skin is not irritated, and cutaneous inflammations of a low type are entirely prevented.

---

**Enemata of Chloral in Sick Headache.**

Dr. J. Seure (*Bull. Gen. de Therap.*, 1878, p. 365,) recommends this treatment very highly. He says that a patient of his, a lady, who is subject to severe attacks of migraine after shopping, etc., is accustomed, on her return home, to take an enema consisting of a glass of warm water, with a tablespoonful of the following mixture: ℞ chloral, gr. xlv.; aq. destillat., f. ℥x.—M. She then reclines upon a a sofa, with closed eyes. Within a few *seconds* she begins to taste the chloral in her mouth, and at the same time she experiences a sensation of numbness. Little by little the headache disappears, nausea is allayed, and half an hour later nothing remains but a slight discomfort in the head, with a little torpor.

Within an hour and a half this lady finds herself able to sit down to dinner, and by the time the meal is over she has forgotten all about her headache and is able to entertain visitors during the evening. In this case twenty grains of the chloral are enough, but in the case of men thirty to forty grains are required. Dr. Seure has noticed that the relief gained is more prompt if a tablespoonful of brandy or whisky is added to the enema. The enema has one disadvantage; that is, the slight burning pain which it causes in the rectum. This may be avoided by the use of a glass of warm milk instead of water, or better by beating up the yolk of an egg in the water. In the case of individuals who retain enemata only with difficulty, a smaller amount may be injected, and a drop or two of laudanum may be added. Dr. Seure regards this treatment as almost infallible for the arrest of an attack of sick headache, and as decidedly preferable to the administration of remedies by the mouth. It has the advantage of not disturbing the stomach. Chloral also acts very promptly, its absorption by the rectum being almost instantaneous, as is proved by the effects on the general system, and also by the exhalation of chloroform by the lungs within a few seconds after the enema has been taken.—*Phila. Med. Times.*

---

**New Method of Covering the Taste of Cod Liver Oil.**

Dr. Ponteres mixes a tablespoonful of cod-liver oil with the yellow of an egg, and when they are thoroughly combined adds to them a few drops of spirits of mint and half a glass of sugar-water. In this way he obtains a sort of mulled egg, which differs very little from ordinary mulled egg, and which presents neither the taste nor the odor characteristic of cod-liver oil. It can consequently be taken without repugnanee by the most fastidious patients.—*Union Med. in Richm. and Louisv. Med. J.*

---

**Spirit of Walnut for Vomiting.**

Ed. Mackey, M.D., M.R.C.P., recommends (*London Practitioner*) the spirit of walnut, made after the following formula, for vomiting: Fresh walnuts, ℥ xxx.; spirit of wine (rect.), ℥ xii.; water. q. s.; distil ℥ xvj. He says: "My own experience is, in general terms, of quick and marked benefit from drachm doses, given every one to four hours, in a little water, for hysterical vomiting, for that of obstinate dyspepsia, and of pregnancy, for anomalous cases, and even for cerebral vomiting. I have tried it in septicæmia, and cannot be surprised if in that case I was disappointed; in the other cases it has always given more or less relief.—*Louisville Med. News.*

---

**Detection of Glucose in Urine.**

Dr. H. G. Piffard offers as a convenient substitute for Fehling's solution the following mixture, which may be kept ready made, and may be carried in a small vial to be used any time at the bedside in the house of a patient: Take of sulphate of copper (C. P.), one part; crystallized tartrate of sodium and potassium, five parts; sodic hydrate (C. P.), two parts. Mix well in a mortar. The resulting mass can be kept in a wide-mouthed bottle until wanted. For use, a piece the size of a pill is dissolved in a couple of drachms of water in a test tube. A few drops of the suspected liquid are now added, when, if sugar be present, the usual reaction will be manifested.

Dr. Lyons recommends the following modification of the copper test for sugar in the urine:

Put into a test tube, in succession, four drops of a solution of pure copper sulphate, 1:10; four drops of a solution of tartaric acid, 1:4; about half a fluid drachm of solution of potassium hydrate, 1:8; boil, and proceed as with Fehling's solution. Made of the strength directed, the solution of tartaric acid is found to keep perfectly well; some glycerine may be added, or a minute quantity of salicylic acid to insure its keeping, or the tartaric acid (about one grain) may be added in the dry state, but the method as described, gives perfectly satisfactory results.

---

**A New Blistering Plaster.**

Mr. G. Dannecy, chief pharmacian of the Bordeaux hospitals, proposes in the *Bulletin de Therapeutique* the following improvement in the mode of applying blisters:

The custom of spreading camphor on the surface of blisters, he remarks, for the purpose of counteracting the internal effects of cantharadine is the cause of frequent deceptions. Every day almost, serious accidents are observed to result from the application of even small blisters, although they had been very carefully camphorated. A more reliable addition he finds to be that of bicarbonate of soda or exsiccated carbonate of soda.

The following is the *modus operandi:* The blistering plaster having been spread of the shape and size indicated, is dusted with a mixture of equal parts of coarsely powdered cantharides and carbonate of soda. The plaster is then strongly pressed with the palm of the hand to insure the adherence of the powder, and the surface is covered with oiled tissue paper. This method has been used exclusively for several years past, and only one complaint has ever been noted, no matter what may have been the size of the blisters. Besides, it has been observed that the vesicating effect was produced more rapidly than with the ordinary plaster.

The author thinks it possible, and even probable, that an alkaline cantharidate is formed. But whatever may be the combination that takes place, the indisputable fact remains that the addition of an alkaline salt prevents the accidents caused by the absorption of cantharidine.

---

**The Best Form of Mercury for Hypodermic Use**

Dr. R. Kirk gives, in the *British Medical Journal*, his experience on this subject while he was at the Edinburgh Lock Hospital. He says:

Six solutions were used during my term of office; the bichloride with morphia; the albuminous solution of the bichloride, Savory and Moore's lamels; a simple aqueous solution of the bichloride; the biniodide, and the bicyanide. The last two were soon discontinued, as possessing no perceptible advantages over the others; but two series of comparative experiments with the other four were made with the utmost care. The first series was conducted to determine which produced the least irritation, and the patients were injected with all four solutions in rotation, with the result, rather surprising, I must admit, of showing that the simple aqueous solution of the bichloride, two grains to the ounce, produced as little irritation as any of the others. In the whole series of over three hundred injections, there was not a single boil, and only on rare occasions was there induration enough to cause tenderness. The second series showed that the amount of mercury required was the same, whatever solution was employed.

The only reasons to which I can ascribe these results are: Thoroughly cleansing the syringe-needle before each injection, and inserting the needle perpendicularly and deeply into the subcutaneous tissue. I believe if these two points are carefully observed, that mercurial injections may be given with almost perfect safety, and with a solution so simple as an aqueous one of the bichloride.—*The Med. and Surg. Reporter.*

---

**For External Use.**

These solutions are kept prepared for use in the New York Hospital:

*Antiseptic Solutions.*

Sol. acid carbolic, 1–20 water
" " " 1–30 "
" " " 1–40 "
" " boraic, 1–30 "
" thymol, 1–1000.

Distilled water is preferred; it will make a clearer solution than ordinary water.

*Carbolic Spray* (Dr. Wheelock).

℞

Sodii bicarb.

Sodi biborat, *aa* ʒ i.

Acidi carbolici, gr. xl.

Glycerini, ʒ vii.

Aquæ, ad. ℥ viii. M.

*White Wash* (Dr. Bulkley.)

℞
Potassii sulphuret.
Zinci sulphat, *aa* ʒ i.
Aquæ, ℥ iv.
Dissolve each in 2 oz. water and mix.

*Red Wash.*

℞

Zinci sulphat, ℈ ii.

Spts. lavand co., *f* ʒi.

Aquæ, O i.

Cochineal coloring, q. s. M.

*Ward Gargle.*

℞

Tannin, ʒ ss.

Sol. potassii chlorat. sat., ℥ viii. M.

M*uriate of Ammonia Wash.*

℞
Ammonii chloridi, ℥ ss.
Tinct. opii, ℥ i.
Aquae, ad O ii. M.

*Lead and Opium Wash.*

℞

Liquor plumbi subacet, ʒ iii.

Aquæ, O i.

Tr. opii, ʒ iv. M.

---

**Croup Cured by Hypodermic Injection of Sulphate of Atropia.**

Dr. De Ponteves, of Antibes, has published (*L' Union Medicale*) a full account of a case of croup, where a fatal termination seemed inevitable, but which resulted in recovery, owing, he believes to hypodermic injections of sulphate of atropia. On the third day of the attack he found his patient—a child three years old—to whom the usual remedies had been given, in a state of commencing asphyxia. The efforts to breathe could be heard in the street; the epigastrium, instead of rising at each respiration, was hollow; the face and neck were enormously swollen and of a violet color; there had been no attempt at vomiting, though large doses of sulphate of copper had been given. At once three drops of a 1 per cent. solution of sulphate of atropine were injected by a Pravaz syringe, on the left side of the neck, on a level with the pneumogastric. At the end of a few minutes a change for the better took place, the respiration became less frequent, and the crowing diminished. Four hours afterwards the child was found tranquil, and, though the respiration was still troubled, dyspnœa was no longer intense. A second injection was given, and the amelioration shortly afterwards became very marked. A few days afterwards the recovery was complete. *A priori* the treatment is a rational one. The real cause of death in croup does not reside in the false membranes.

M. Jacoud, in his *Traite de Physiologie Interne*, speaks of the "very numerous cases in which croup kills without laryngeal obstruction sufficient to explain death." He adds that, "though often the expulsion of the false membrane is followed by great relief marking the diminution of the dyspnœa, yet the cases are far from rare in which the remission is absent or inappreciable—a fact sufficient to prove that croupal dyspnœa has more causes than the obstruction of the larynx by exudation." When the pneumogastric nerves of dogs are divided in the neck, what always happens is the occlusion of the glottis from paralysis of the recurrents, and after death there is found intense congestion of the lungs, pulmonary œdema, dilation of the smaller bronchi, and vesicular emphysema. Now these symptoms and lesions are also observable in croup and capillary bronchitis. The essential cause, therefore, of asphyxia in croup seems to be the paralysis—more or less complete—of the pneumogastric. This view is supported by the fact that it is difficult, and often impossible, to produce vomiting. Belladonna, being an excitant specially of the pneumogastric, appears to be indicated in cases such as have been detailed.—*Dublin Journal of Medical Science.*

---

**Salicylate of Soda in Acute Rheumatism.**

Dr. Blackwood, of Philadelphia, writes to the *Medical Record* the following correcting statement. It is corroborative of a thousand similar reports. He says:

"In a recent number of your journal, Professor Stille makes the remarkable assertion that '*no* treatment was ever invented that stopped a case of articular rheumatism,' and that therefore the cure may be abandoned 'to nature, aided by palliatives.' Had this statement been enunciated thirty years ago, little would have been thought of it; at this day a good deal will not only be thought about it, but probably written on the subject. My attention was early drawn to this painful affection from its prevalence in my own family; and the plan of leaving the cure to nature in a second attack, by the skillful physician who then attended our family, resulted in the death of the patient from endocarditis. Having shortly after the time alluded to, engaged in practice myself, I treated another member of my family suffering with an extremely acute attack, and succeeded in decidedly shortening the period of his confinement to bed. The plan then in vogue—now nearly twenty years since—was crude as compared with the facilities we now possess, yet notable success always ensued in all cases *promptly and thoroughly* treated. The fact that various methods were urged by different authorities did not at that time militate against their value, for rheumatism, as any other disease, may be relieved or cured by more than one plan of treatment. After seeing a fair amount of rheumatic fever during nine years of my army life, I came back to this city in time to find the second case referred to convalescing from a bad attack which lasted *thirty-seven* days under 'expectant' treatment in the hands of a well known Walnut street practitioner. The next attack set in with violent symptoms, intense pain in all the articulations of the lower extremities, and in nearly all those of the upper extremities. I saw him twenty-four hours after taking to bed—his temperature $102\frac{6}{10}°$, rapidly mounting to $104°$, before remedies could be thoroughly applied. There is no necessity for going into the daily symptoms; so much has already been published concerning salicylic acid as to make repetition useless. Suffice it to say that, under twenty-grain doses of salicylate of sodium, in forty-eight hours the temperature was reduced to the normal point, the pain was gone, the inflamed joints were free from swelling, not productive of agony when handled, and the patient, who was anxious to return to his business as soon as possible, was out of the house in seven days. More than that, he has never since then (1875), had a touch of his old enemy. This case is one out of many, and with uniform success in upward of a hundred cases since that time. Much as I respect the opinions of the distinguished Professor, I cannot help differing decidedly and strongly from his statement. The experience of physicians in many thousand cases has proved the value beyond question of the salicylates, and in closing I do not hesitate to say that in the salts mentioned we possess against rheumatism as decided a specific, and more so, than we do in quinia against the so-called malarial fevers."

